# Prospective use of wastewater surveillance for early detection of enterovirus D68 in community outbreaks among children, Niigata City, Japan, 2024

**DOI:** 10.2807/1560-7917.ES.2026.31.20.2500779

**Published:** 2026-05-21

**Authors:** Jun Tachikawa, Yuta Aizawa, Rie Habuka, Kotaro Tsushima, Nur Irma Safitri, Tatsuki Ikuse, Masaaki Kitajima, Akihiko Saitoh

**Affiliations:** 1 Department of Paediatrics, Niigata University Graduate School of Medical and Dental Sciences, Niigata, Japan; 2 Department of Paediatrics, Niigata City General Hospital, Niigata, Japan; 3 Research Center for Water Environment Technology, Graduate School of Engineering, The University of Tokyo, Tokyo, Japan

**Keywords:** Wastewater surveillance, Enterovirus D68, Outbreak, Children, Japan

## Abstract

**INTRODUCTION:**

Enterovirus D68 (EV-D68) is a re-emerging pathogen that can cause acute respiratory illness in children and difficult to predict outbreaks.

**AIM:**

To anticipate outbreaks in children, we investigated if wastewater surveillance could timely detect circulation of EV-D68 and its respective subclades in Niigata City, Japan.

**METHODS:**

During January−December 2024, wastewater samples were collected once a week for EV-D68 RNA concentration determination. Numbers of children (≤ 15 years old) admitted with wheezing to six hospitals with paediatric beds in Niigata City were monitored; in a subset of children, nasopharyngeal swabs were tested for EV-D68 during September−December. Nucleotide sequences of EV-D68 derived from patient and wastewater samples were phylogenetically analysed.

**RESULTS:**

During January−December 2024, EV-D68 RNA was found in 39 (41.9%) of 93 wastewater samples. The first EV-D68 detection occurred in week 27, with RNA concentrations rising in subsequent samples. From week 32, numbers of children hospitalised with wheezing increased, peaking in week 37. Up to week 52, 195 children with wheezing were admitted to the six hospitals. The wastewater EV-D68 RNA concentrations positively correlated with paediatric hospitalisations for wheezing (ρ = 0.54, p < 0.001). In weeks 37–52, 16 (10 girls/6 boys; median age: 4.8 years; interquartile range: 3.1–7.5) of 31 hospitalised children tested for EV-D68 were positive. Strains affecting them were of subclade B3 only, while strains in wastewater belonged to subclades D1 and B3.

**CONCLUSIONS:**

Wastewater surveillance timely detected an increase in EV-D68 activity, revealing the circulation of subclades undetected among patients. It thus can potentially support EV-D68 outbreak prediction and response and could possibly be extended to other pathogens.

**Key public health message**

**What did you want to address in this study and why?**

Enterovirus D68 (EV-D68) is a pathogen with different subclades. It can cause recurring epidemics of acute, sometimes severe, respiratory illness in children. As predicting EV-D68 outbreaks is difficult, we wanted to see if detections of EV-D68 in wastewater allowed anticipating rises in paediatric hospitalisations for wheezing in Niigata City, Japan. We also aimed to determine the EV-D68 subclades occurring in wastewater and affecting patients.

**What have we learnt from this study?**

We learnt that amounts of EV-D68 RNA in wastewater rose approximately 5 weeks before increases in paediatric hospital admissions for wheezing occurred. Wastewater EV-D68 concentrations correlated with hospitalisation trends. While strains detected in patients were of subclade B3 only, strains in wastewater samples were both of subclade B3 and D1, suggesting circulation of additional, undetected subclades in the community.

**What are the implications of your findings for public health?**

Wastewater surveillance can provide a timely warning of EV-D68 outbreaks, enabling earlier preparation of healthcare systems and targeted clinical testing. It also helps reveal viral diversity in the community beyond what is detected in patients. Incorporating wastewater monitoring into routine public health surveillance may improve outbreak prediction and response, not only for EV-D68 but also for other respiratory pathogens.

## Introduction

Enterovirus D68 (EV-D68) belongs to EV species D in the *Picornaviridae* family. It can lead to asthma-like respiratory illness in children and is epidemiologically associated with acute flaccid myelitis [1]. While cough and wheezing are the hallmark symptoms of severe EV-D68 infection in paediatric patients, the virus can also cause mild acute respiratory illness (ARI), such as mild cough and rhinorrhoea [2]. Paediatric populations, particularly those under the age of 5 years, represent the group at highest risk for severe EV-D68 infection [3]. Although children with asthma are at high risk of severe EV-D68 infection, EV-D68 can cause severe disease in otherwise healthy children of all ages [2]. In contrast, while healthy adults can be infected with EV-D68, they usually present with mild symptoms or even no symptom [2].

Following its initial isolation in 1962, EV-D68 was first only sporadically detected. However, in 2014, a large outbreak of EV-D68 with severe respiratory infections in children was reported in the United States (US) [2]. Since, outbreaks of the virus have been recognised in several countries. Notably, EV-D68 typically follows a biennial circulation pattern, with, in temperate regions, peaks in late summer and autumn [1]. The virus is genetically diverse and classified into four clades (A–D) based on the sequence of the viral protein 1 (VP1) region [1]. Clades A and B are further divided into subclades A1 and A2 and B1, B2, and B3, respectively. Subclade A2 is further subdivided into D1 and D2 (also known as A2/D1 and A2/D2, respectively) [3]. Starting 2016, clade B3 has emerged as the globally dominant clade in the US [4], Europe [3], Asia [5,6] and Africa [7,8]. Additionally, clade D1 has been detected since 2018 in Europe [9].

In Japan, cases of paediatric asthma-like respiratory illness and acute flaccid myelitis notably increased in 2015, in association with an EV-D68 outbreak [10,11]. In 2018, we reported a significant increase in EV-D68-associated asthma-like respiratory disease among children in Niigata Prefecture, Japan [5]. Although the observed periodicity suggested that another outbreak would occur around 2021, a considerable resurgence was not noted in 2019–2023, potentially because of the impact of the COVID-19 pandemic and associated public health measures. Prediction of the next EV-D68 outbreak is therefore a challenge. 

As part of the nationwide pathogen surveillance under the Infectious Diseases Control Law in Japan, laboratories at the Prefectural Institutes of Public Health (the public health laboratories) examine clinical samples originating from clinics and hospitals in each prefecture. For patients with ARI, acute flaccid paralysis, or acute flaccid myelitis, clinical samples include nasopharyngeal (NP) swabs and cerebrospinal fluid. These samples are sent to the laboratories when analyses are requested by physicians.

Wastewater surveillance is used to monitor community health by analysing sewage for pathogens and other substances [12]. Wastewater surveillance is a non-invasive, population-level approach for capturing data from both symptomatic and asymptomatic individuals and is now used to detect respiratory pathogens, such as severe acute respiratory syndrome coronavirus 2 (SARS-CoV-2) [12,13], respiratory syncytial virus (RSV) and influenza viruses [12]. However, its application to non-polio EVs, such as EV-D68, is limited. In January 2024, we initiated wastewater surveillance for respiratory viruses, including EV-D68, in Niigata City, Japan. In the present study, we aimed to investigate if wastewater surveillance of EV-D68 can be used to timely predict EV-D68 outbreaks in children and to identify the viral subclades having the potential to cause these outbreaks.

## Methods

### Wastewater data

#### Wastewater sample collection

**Figure 1:**
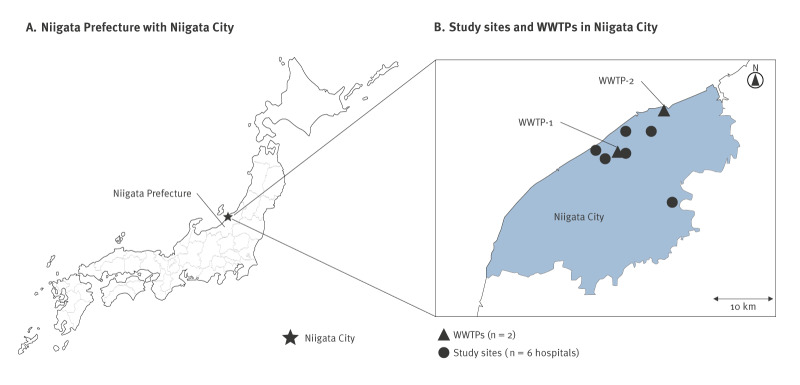
Locations of (A) the Niigata Prefecture including Niigata City and (B) the six hospitals and two WWTPs studied in Niigata City, Japan, 2024

Wastewater samples were collected from two wastewater treatment plants (WWTPs) in Niigata City (WWTP-1 and WWTP-2) (Figure 1), which are the largest among the city’s eight WWTPs in terms of service population. The number of residents served by WWTP-1 and WWTP-2 are 228,213 and 188,644, respectively [14,15], corresponding to 29.8% and 24.6% of the city’s total population of 766,797. Of the remaining population, 12.8% (n = 98,431) are not served by any WWTP, while 32.8% (n = 251,509) are served by the other six WWTPs. The WWTP-1 serves a predominantly combined sewer system (75.5% combined and 24.5% separate), while the WWTP-2 serves a predominantly separate sewer system (95.4% separate and 4.6% combined) [14,15]. The data on the average dry weather flow and peak wet weather flow of the two WWTPs in 2024 are summarised in Supplementary Table S1.

WWTP: wastewater treatment plant.

Niigata City is the capital of the Niigata Prefecture.

Untreated influent wastewater samples were collected by grab sampling once a week, on either Wednesday or Thursday from both WWTPs. Sampling at WWTP-1 was conducted from January (week 2) through December (week 52), 2024, and at WWTP-2 from February (week 5) through December (week 52), 2024. Wastewater samples (500 mL) were collected from each plant in sterile plastic bottles and transported on ice to the laboratory at Niigata University within 4 hours of collection.

#### Detection of EV-D68 RNA in wastewater samples

Viral ribonucleic acid (RNA) in wastewater was detected using the Efficient and Practical virus Identification System with ENhanced Sensitivity for Membrane (EPISENS-M) method [13]. Briefly, 300 mL of wastewater supplemented with a final concentration of 25 mM MgCl_2_ was filtered through electronegative membrane (90-mm diameter; 0.8-μm pore size; Merck Millipore, Billerica, Massachusetts, US). Filtration was conducted within 8 hours after sampling, and the membranes were stored at − 80 °C until further analysis. A quarter of the membrane was subjected to RNA extraction using a PowerWater bead beating tube (RNeasy PowerWater Kit; Qiagen, Hilden, Germany), QIAcube Connect platform, and RNeasy PowerMicrobiome Kit (Qiagen).

The RNA was then subjected to one-step reverse transcription followed by preamplification (RT-Preamp) using the iScript Explore One-Step RT PreAmp Kit (Bio-Rad Laboratories, Hercules, California, US), with EV-D68-specific primers from the real-time polymerase chain reaction (PCR) assay previously developed by our laboratory, targeting the genetic region with the sequence encoding the virus VP1 [16]. The RT-Preamp product was then subjected to real-time PCR. While the previous real-time PCR protocol [13] used One-Step PrimeScript RT-PCR (TaKaRa, Shiga, Japan) directly from RNA, this study employed iQ Supermix (Bio-Rad Laboratories) because the templates used for the final detection were pre-amplified complementary (c)DNA rather than RNA. This modification was necessary to optimise the quantification of the target cDNA following the RT-Preamp step. Accordingly, the thermal cycling conditions were optimised for cDNA templates. The revised conditions consisted of an initial denaturation at 95 °C for 3 min, followed by 45 cycles of 95 °C for 15 s and 55 °C for 45 s.

Pepper mild mottle virus (PMMoV) RNA was used to normalise EV-D68 RNA concentrations. This normalisation was performed specifically to account for the dilution effects caused by environmental factors, such as heavy rainfall and seasonal snowmelt, which can influence wastewater flow rates and viral concentrations. The real-time PCR for PMMoV was performed as previously reported [13], except the One-Step PrimeScript RT-PCR kit (TaKaRa) was used instead of the quantiTect Probe PCR Kit (Qiagen), and thermal cycling conditions were as follows: 42 °C for 5 min, followed by 95 °C for 3 min, and 45 cycles of 95 °C for 5 s and 60 °C for 40 s.

All real-time PCR reactions were performed with the Thermal Cycler Dice Real Time System III (TaKaRa). Viral RNA was quantified by real-time PCR with a standard curve generated from serial dilutions of gBlocks (Integrated DNA Technologies, Coralville, Indiana, US) for EV-D68 and PMMoV. The limit of detection for EV-D68 RNA of the EPISENS-M method was 1 copy/μL of RNA, which corresponds to 6.7 × 10^2^ copies/L of wastewater, under the assumption that the recovery efficacy of viral RNA for the filtration and RNA extraction process was 100%. The EV-D68 RNA concentration was normalised by dividing it by the concentration of PMMoV in the same sample.

### Clinical data

#### Case definitions

In this study, we focused on the hospitalised patients with wheezing, as wheezing is one of the most common clinical manifestations of infection with EV-D68. We defined ‘paediatric patients with wheezing’ as children (age ≤ 15 years) presenting with wheezing and diagnosed with asthma-like respiratory illness or asthma exacerbation by paediatricians.

#### Nasopharyngeal swab sampling from paediatric patients with wheezing

In early September 2024 (week 37), paediatricians in Niigata City hospitals observed an apparent increase of hospitalisations concerning children with wheezing. This led to the prospective collection of NP swabs from such paediatric inpatients, who consented to participate in the study until  week 52, 2024. The locations of the six hospitals are shown in Figure 1. Using the medical records of each hospital, we also collected data on age, sex (collected as a binary variable: male or female) of the patients, past medical history of asthma or wheezing episodes, days from symptom onset to admission, symptoms, diagnosis, treatment, intensive care unit management, and length of hospital stay. The presence of respiratory pathogens other than EV-D68, such as RSV and influenza virus, was evaluated by rapid antigen tests. Infection with SARS-CoV-2 was assessed by rapid antigen testing or PCR. Other respiratory pathogens were tested with the FilmArray respiratory panel (BioFire Diagnostics, Inc., Salt Lake City, Utah, US). The decision to perform these tests was made by the paediatricians in each hospital. Patients with positive results for pathogens other than EV-D68 were excluded from this study. Cases with co-detection of EV-D68 and other pathogens were also excluded. This approach was adopted to specifically evaluate the clinical impact and seasonal dynamics attributable solely to EV-D68.

#### EV-D68 detection in clinical specimens

Admitted paediatric patients’ NP swabs were sent to the laboratory of Niigata University on ice and stored at − 80 °C until analysis. This laboratory is a research-based laboratory testing clinical samples from paediatric patients in certain circumstances (from tertiary care, severe, or complicated infections). Extraction of RNA from samples was conducted with the QIAamp minElute Virus Spin Kit (Qiagen) in accordance with the manufacturer’s instructions. Real-time PCR and semi-nested PCR assay was used to detect EV-D68, as previously described by our laboratory [16].

### Sequencing and phylogenetic analysis

#### Sequencing for wastewater samples

For wastewater samples testing positive for EV-D68 by the EPISENS-M method, the viral RNA extract was subjected to cDNA synthesis using Super-Script VILO MasterMix (Invitrogen), followed by the semi-nested PCR to amplify EV-D68 partial VP1, as previously described [16]. When the band of target-size was observed in the gel electrophoresis, the semi-nested PCR products were analysed by Sanger sequencing and genotypes were determined based on phylogenetic analysis. When overlapping peaks were observed in the Sanger sequencing chromatograms, TOPO cloning (Thermo Fisher Scientific) was performed as previously described [17], with the exception that the semi-nested PCR products were purified by using NucleoSpin Gel and PCR Clean-up (TaKaRa) in accordance with the manufacturer’s instructions.

#### Sequencing for clinical samples

For all real-time PCR-positive samples, semi-nested PCR was performed, and products were analysed by Sanger sequencing. Additionally, we subjected real-time PCR-negative samples to the semi-nested PCR and sequencing, because the real-time PCR assay may have lower sensitivity for Clade D [16].

#### Phylogenetic analysis

In addition to the sequence data obtained by sequencing wastewater samples and clinical samples, nt sequence data for other EV-D68 strains, which were reported as detected in the period 2014–2024 were obtained from GenBank for phylogenetic analysis. Using the Molecular Evolutionary Genetics Analysis software, version 6 [18], after multiple alignments with the Clustal W programme, we identified the best substitution model, as indicated by the lowest Bayesian information criterion scores. A phylogenetic tree was constructed by using the maximum likelihood method with the best substitution model with 1,000 bootstrap replicates. We also performed sequence similarity searches against the National Center for Biotechnology Information (NCBI) GenBank database using nt Basic Local Alignment Search Tool (BLASTn; https://blast.ncbi.nlm.nih.gov/Blast.cgi).

### Weekly number of admitted paediatric patients with wheezing

We retrospectively collected data on the weekly number of paediatric patients with wheezing admitted to the six Niigata City hospitals during the period from 1 January 2024, through 31 December 2024. The proportion of children aged 15 years or younger in Niigata city was 12.2% (93,387/766,797) as of 2024 [19].

### Statistical analysis

Spearman’s rank correlation test was used to assess the association between PMMoV-normalised concentrations of EV-D68 RNA in wastewater and weekly numbers of admitted paediatric patients with wheezing. A two-sided p value of < 0.05 was considered to indicate statistical significance. All statistical analyses were conducted with R version 4.4.1 [20].

## Results

### Trends in wastewater EV-D68 RNA concentrations and weekly number of admitted paediatric patients with wheezing

**Figure 2:**
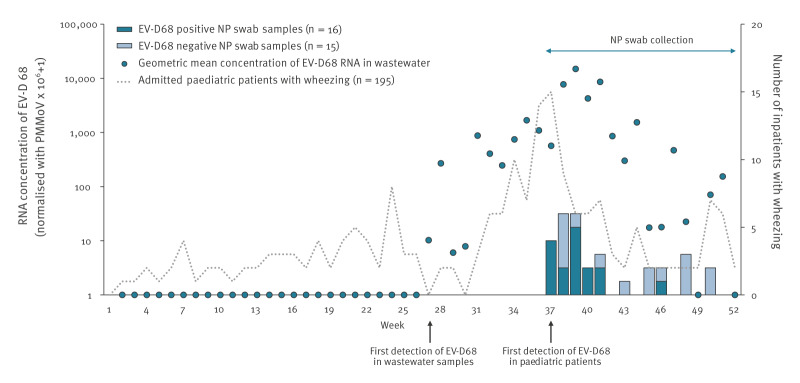
Weekly concentrations of EV-D68 RNA in wastewater samples^a^ and weekly numbers of paediatric patients experiencing wheezing admitted to hospital^b^, with those who were tested for EV-D68^c^, stratified by positive or negative result, Niigata City, Japan, 2024

EV-D68: enterovirus D68; NP: nasopharyngeal; PMMoV: pepper mild mottle virus.

^a ^The samples were obtained from two WWTPs.

^b ^The children were admitted to six hospitals.

^c^ Patients who were tested for EV-D68 through an NP swab were among those admitted during weeks 37–52.

From January through December 2024, a total of 93 wastewater samples were collected (45 from WWTP-1 and 48 from WWTP-2). In 39 (41.9%) of the 93 wastewater samples, EV-D68 was detected by the EPISENS-M method employing real-time PCR (18 of 45 samples from WWTP-1 and 21 of 48 samples from WWTP-2). The first detection of EV-D68 from wastewater occurred in week 27 of 2024. In wastewater, PMMoV-normalised EV-D68 concentration increased until reaching a peak in week 39 and EV-D68 RNA was detectable until week 51 (Figure 2), as also described in Supplementary Table S2.

The black circles indicate the geometric mean concentrations of EV-D68 RNA in wastewater from two wastewater treatment plants (normalised with concentrations of PMMoV). Data are presented on a logarithmic scale. To facilitate the plotting of zero or undetectable values, a constant of 1 was added to all data points. The dotted line indicates the weekly number of children with wheezing who were admitted to six hospitals in Niigata City, Japan, in 2024. During weeks 37–52, NP swab samples were collected from the admitted patients. The dark blue bars indicate the weekly number of positive samples for EV-D68 in NP swabs from children with wheezing, as determined by real-time PCR. The light blue bars indicate the number of EV-D68-negative samples. No sample that was negative on the real-time PCR assay was positive on partial VP1 semi-nested PCR. In wastewater samples and paediatric patient samples, EV-D68 was first detected in week 27 and week 37, respectively.

From January through December 2024, 195 paediatric patients with wheezing were admitted to the six hospitals in Niigata City, Japan. The average weekly number of admissions was 3.8 cases per week, and the peak number was 15 cases per week, in week 37 (Figure 2). The weekly mean concentrations of EV-D68 RNA in wastewater from the two WWTPs positively correlated with the weekly number of admitted patients (ρ* =* 0.54, p < 0.001).

### Detection of EV-D68 in nasopharyngeal swabs from admitted paediatric patients with wheezing

Our routine testing protocol did not include EV-D68 for children who required hospitalisation for wheezing, however, an increase in paediatric hospitalisations with wheezing was identified in early September 2024 (week 37), as indicated by personal communications with paediatricians in Niigata City hospitals. Collection of NP swabs was thus started on 13 September 2024 (week 37) and continued until 29 December 2024 (week 52). Among the 78 admitted children with wheezing during weeks 37–52 of 2024, NP swabs were collected from 31 patients (39.7%). Weekly observation showed that patients were positive for EV-D68 during weeks 37–41, with a peak in week 39 (Figure 2). Real-time PCR testing revealed that 16 of 31 patients were EV-D68 positive, while 15 of 31 had negative results. In the second screening of the 15 patients with negative results by the real-time PCR assay, no samples were positive for VP1 by semi-nested PCR. The median age of EV-D68–positive patients was 4.8 years (interquartile range: 3.1–7.5 years), and 10 of the patients were female, while six were male. Symptoms other than wheezing, treatment and length of hospital stays are summarised in Supplementary Table S3.

### Genotyping and phylogenetic analysis

The EV-D68-specific VP1 sequence was successfully identified in 14 of 16 real-time PCR-positive clinical samples. Among the 39 EV-D68-positive wastewater samples, VP1 amplification and sequencing was successful for 19, but the VP1 region could not be amplified for 12. For the remaining eight samples, amplification was successful but overlapping peaks were observed in the Sanger sequencing results. Cloning of the PCR amplicons enabled successful sequencing in four of these eight samples.

**Figure 3:**
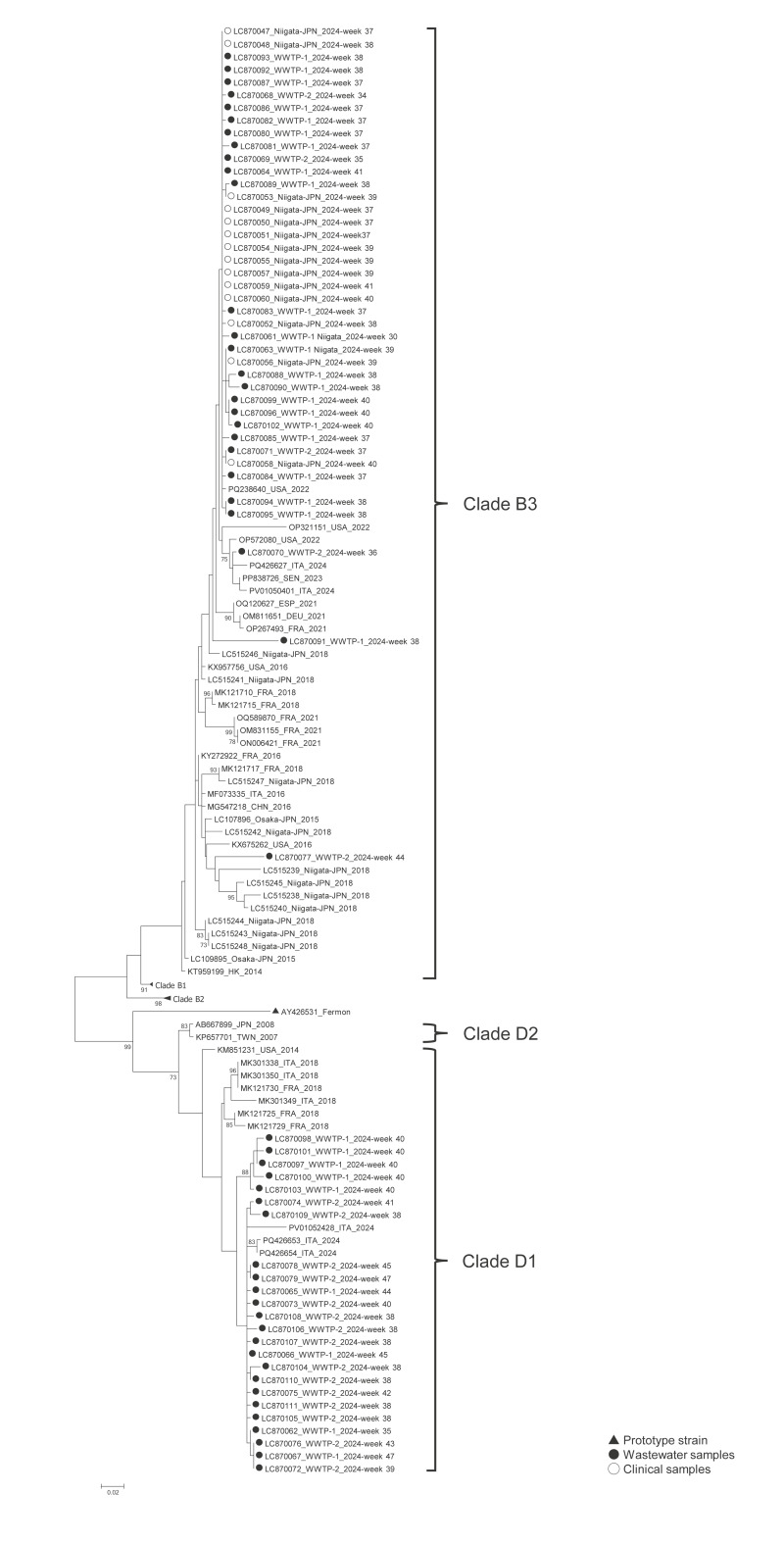
Phylogenetic analysis results of EV-D68 strains detected in samples from paediatric patients (n = 14) and wastewater (n = 23), based on nt sequences covering the partial VP1 region of the EV-D68 genome, Niigata City, Japan, 2024

EV-D68: enterovirus D68; VP1: viral protein 1.

**Figure 4:**
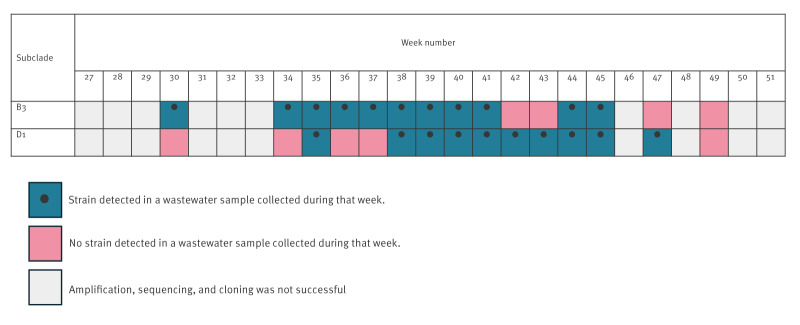
Weekly distribution of EV-D68 strains detected in wastewater in Niigata City, Japan, 2024

Phylogenetic analysis of the partial VP1 sequence revealed that all EV-D68 strains detected from NP swabs were in subclade B3, while strains detected from wastewater samples were in two subclades: B3 and D1 (Figure 3). The subclade B3 strains detected in wastewater and clinical samples in this study shared 92–99% sequence identity with the recent globally dominant B3 strains reported from the US (GenBank accession number: OP321151), Europe (PQ426627) [21] and Asia (MG547218). Similarly, the strains in subclade D1 showed 95–99% identity to the reference strains in Europe (PQ426654, MK121729) [9,21]. Strains in clade B3 and clade D1 were detected from wastewater samples in weeks 30–45 and in weeks 35–47, respectively (Figure 4).

The nt sequences of the partial VP1 region were analysed, and a phylogenetic tree was constructed with Molecular Evolutionary Genetics Analysis software, version 6, using the maximum likelihood method with the Tamura 3-parameter and discrete Gamma distribution with 1,000 bootstrap replicates. The numbers on each branch indicate the bootstrap values (with those above 70% shown). The scale bar shows nt substitutions per site. Data from nt sequences of other EV-D68 strains detected in reports for the period 2014–2024 were downloaded from the GenBank database. The black triangle indicates the prototype strain. Black circles and white circles indicate strains from wastewater samples and clinical samples in Niigata City, respectively. Strains from GenBank are recorded as ‘accession number_detected region_detected year’. Strains from clinical samples are recorded as ‘accession number_detected region_detected year-week’, and strains detected in wastewater are recorded as ‘accession number_detected wastewater treatment plant (WWTP)_detected year-week’.

EV-D68: enterovirus D68

EV-D68 was not detected in week 49. Amplification, sequencing, and cloning was not successful for samples collected in weeks 27–29, 31–33, 46, 48, 50, and 51, despite the detection of EV-D68 RNA in these samples.

## Discussion

Our findings show that wastewater surveillance provided an early warning of an EV-D68 activity increase in Niigata City, Japan, in 2024, 5 weeks before paediatric hospitalisations for wheezing rose. Capturing EV-D68 outbreaks based on current clinical surveillance is challenging. Indeed, the six hospitals in this study do not routinely screen for EVs among paediatric patients with respiratory symptoms. Moreover, the detection of EV-D68 by real-time PCR analysis requires specimens to be submitted to the university laboratory in Niigata City, a research specialised laboratory that does not process all routine clinical samples in Niigata City/Prefecture. 

Within Japan, the integration of clinical and environmental surveillance data is primarily conducted on a research or pilot basis in a few cities. In the country, EV-D68 is included in a passive pathogen surveillance that captures a variety of respiratory pathogens but is not specifically targeted at this virus. While some hospitals employ a multiplex PCR panel to detect respiratory viruses, which can identify EVs/rhinovirus, this lacks specificity for EV-D68. Thus, the detection of EV-D68 requires specimens to be submitted to public health laboratories. As a result, the surveillance likely underestimates its true prevalence. Moreover, the public health laboratories results, which are published on a prefectural basis, require a few weeks to obtain. 

In contrast, our university laboratory can detect and sequence EV-D68 from wastewater samples within a week. Detection of EV-D68 in wastewater samples in week 27 (July), followed by an increase in hospital admissions of children with wheezing, prompted testing of patients admitted to Niigata University Hospital. This led to the identification of the first paediatric case of EV-D68-associated wheezing in week 37 (September) in 2024, demonstrating the potential of wastewater surveillance to timely warn on a possible EV-D68 outbreak without the need for continuous clinical testing throughout the year. The outbreaks of EV-D68 typically occur every 2 to 3 years, so continuous testing of all symptomatic paediatric patients is not cost-effective. Furthermore, if we initiate patient testing after the detection of EV-D68 RNA in wastewater, we can identify cases more efficiently. Early detection of EV-D68 from wastewater enables us to diagnose EV-D68 cases which are potentially missed in the clinical surveillance.

Since the beginning of the COVID-19 pandemic, wastewater surveillance has become a practical and globally adopted tool for early detection of various pathogens in the community, including EV-D68. Wastewater surveillance for EV-D68 has been implemented and studied in the US [22,23], United Kingdom (UK) [24], Belgium [25], France [26], and Israel [27,28], and these previous investigations also reported early detection of EV-D68 outbreaks [22,23,25,28]. However, prospective use of wastewater surveillance data to directly inform clinical practice has been limited. A study in Israel reported earlier detection of EV-D68 in wastewater than in clinical samples, although the data were analysed retrospectively [28]. Unlike previous studies, we collected clinical data and samples from patients prospectively after a considerable increase in EV-D68 in wastewater was observed. Our findings are the first to show that wastewater surveillance data can potentially lower the threshold for EV-D68 testing and are therefore valuable for clinicians assessing whether to test for EV-D68 in patients with wheezing. In addition, prospective collection of clinical data allowed us to acquire more accurate information on the potential EV-D68 outbreak.

Wastewater surveillance offers critical public health advantages. First, it aids clinicians in performing timely and appropriate assessments of children with wheezing or acute flaccid paralysis/myelitis. By identifying periods of active viral circulation, wastewater surveillance also provides essential data on the pre-test probability of infection in the community. During periods of confirmed wastewater detection, the positive predictive value (PPV) of clinical testing results is likely to increase, allowing for a more targeted and efficient diagnostic approach. This mitigates the need for extensive and potentially unnecessary investigations into other common causes of wheezing, such as typical asthma triggers, including RSV, or allergy. Universal screening of all children with wheezing is not feasible because the outbreaks usually occur every 2–3 years, and screening for all children is costly, labour intensive and invasive for children. Therefore, wastewater data can be a valuable tool for deciding whether to initiate clinical testing for EV-D68. Second, early warning allows diagnostic laboratories to prepare proactively for increased demand for PCR tests, thus ensuring sufficient testing capacity and reagent availability. Third, healthcare systems can utilise this early information to proactively manage resources in preparation for potential surges in emergency department visits and hospitalisations by securing adequate bed capacity and ensuring availability of necessary medications for managing respiratory distress.

Our genetic analysis of the wastewater samples identified circulating EV-D68 strains. In the previous studies in Israel, clade B strains were detected in paediatric respiratory samples and wastewater samples collected in 2014 and 2021 [27,28]. In the UK, in 2021, co-circulation of strains within clades B3 and D was suggested from strains identified in wastewater samples, although clinical samples were not included in that study [24]. In France, between 2014 and 2015, strains detected in clinical samples belonged to subclades A2, B1, and B2; however, only subclades B1 and A2 were detected in wastewater [26]. In the present study, EV-D68 strains in wastewater samples were classified into subclades B3 and D1, although only subclade B3 was detected in samples from paediatric patients. Previous studies analysing only clinical samples reported co-circulation of subclades D1 and B3, beginning in 2018, in France [9], the UK [29], and Spain [30]. In 2024, which is the same year as the current study, subclade B3 and A2/D1 were co-circulating in northern Italy [21]. Notably, previous studies revealed an age-specific distribution pattern, with subclade B3 predominantly isolated from children and subclade D1 more frequently detected in adults [9,21,29,31,32]. Therefore, the discrepancy in clades between strains detected in clinical and wastewater samples suggests that both clades B3 and D1 circulated in Niigata City during the study period; however, strains in subclade B3 might have been more likely to cause respiratory disease in children than those in subclade D1. Thus, our findings highlight the importance of augmenting wastewater surveillance with sequencing and phylogenetic analysis, to better predict the disease burden in a community and understand EV-D68 circulation patterns within that community.

This study has limitations that warrant mention. The collection of NP swab samples from children admitted with wheezing did not begin until week 37 of 2024, thus hindering precise determination of the start of the EV-D68 seasonal outbreak in 2024. The NP swabs were moreover only obtained from the paediatric population, which limits assessment of the circulation of subclade D1 strains in adults. Consequently, it remains unclear whether subclade D1 circulated primarily or exclusively in adults during the study period. Additionally, wastewater samples were not analysed by the semi-nested PCR assay, which has higher sensitivity to clade D compared to the real-time PCR assay, so clade D strains in wastewater may have been missed during the real-time PCR negative period (week 2–26, 49, 52). It should be noted as well that sequencing EV-D68 from wastewater presents some challenges due to low viral loads and genome fragmentation, which restrict the ability to perform comprehensive phylogenetic comparisons between clinical and environmental strains. Also, our clinical analysis focused exclusively on the paediatric population and the sample size of the study was small. Recent reports from EV-D68 outbreak in Europe in 2024 suggested an increasing trend of viral circulation among adults [21]. Since wastewater signals reflect the shedding from the entire community including adults and children, the exclusion of adult clinical cases may explain some discrepancies in our correlation analysis. Future studies should incorporate adult clinical data to achieve a more comprehensive understanding of EV-D68 epidemiology. Furthermore, our case definition was limited to hospitalised paediatric wheezing and excluded patients with co-infections. While this approach focused on severe EV-D68-specific outcomes, it may have underestimated the total clinical cases contributing to the environmental viral load. This exclusion could potentially weaken the correlation between wastewater and clinical data. Last, wastewater samples were collected from only two WWTPs in Niigata City (covering 54.4% of the total population) and therefore do not provide complete coverage of the city population.

## Conclusion

In Niigata City, Japan, wastewater surveillance enabled the early detection of an EV-D68 outbreak among children. Continuous surveillance incorporating both clinical and wastewater tests, expansion to other geographical areas, and the development of predictive models are needed to establish effective early warning systems for EV-D68 outbreaks. Additionally, incorporating wastewater monitoring into routine public health surveillance may improve outbreak prediction and response, not only for EV-D68 but also for other pathogens.

## Data Availability

The nucleotide sequences of the partial VP1 region obtained in this study have been deposited in the DDBJ/EMBL/GenBank databases under accession numbers LC870047 to LC870111.

## Supplementary Data

252000 Supplementary Material
