# Supplementary material for: Prospective use of wastewater surveillance for early detection of enterovirus D68 in community outbreaks among children, Niigata City, Japan, 2024
Source: Euro Surveill. 2026 May 21;31(20):2500779. doi: 10.2807/1560-7917.ES.2026.31.20.2500779 (PMC13197737; doi:10.2807/1560-7917.ES.2026.31.20.2500779)
Supplement: Supplementary Material [file 25-00779_SAITOH_Supplement.pdf]

This supplementary material is hosted by *Eurosurveillance* as supporting information alongside the article 'Prospective use of wastewater surveillance for early detection of enterovirus D68 in community outbreaks among children, Niigata City, Japan, 2024', on behalf of the authors, who remain responsible for the accuracy and appropriateness of the content. The same standards for ethics, copyright, attributions and permissions as for the article apply. Supplements are not edited by *Eurosurveillance* and the journal is not responsible for the maintenance of any links or email addresses provided therein.

Table S1. Average Dry Weather Flow (ADWF) and Peak Wet Weather Flow (PWWF) in the two wastewater treatment plants (WWTP) in Niigata City, Japan, 2024

WWTP	Influent flow	Month											
		Jan	Feb	Mar	Apr	May	Jun	Jul	Aug	Sep	Oct	Nov	Dec
WWTP-1	ADWF (m^3/day)	181,152	143,486	142,950	142,810	141,961	140,195	162,530	149,264	144,728	135,570	141,478	171,825
	PWWF (m^3/day)	509,199	436,709	485,521	488,348	576,371	390,023	858,217	244,390	810,435	385,612	687,365	472,032
WWTP-2	ADWF (m^3/day)	61,199	56,895	56,848	57,352	56,934	58,266	62,368	58,175	60,090	58,005	56,136	62,869
	PWWF (m^3/day)	86,858	70,877	67,558	70,453	72,528	66,044	99,712	61,820	94,248	74,863	93,245	85,282

Table S2. Real-time Polymerase Chain Reaction Ct values and Copy Numbers of Enterovirus D68 ribonucleic acid in wastewater collected from two WWTPs in Niigata City, Japan, 2024

Week	WWTP-1		WWTP-2		WWTP-1 + WWTP-2
	Ct value	Copies/L (normalized with PMMoV $\times 10^6$)	Ct value	Copies/L (normalized with PMMoV $\times 10^6$)	Geometric mean, copies/L (normalized with PMMoV $\times 10^6$)
1	NA	NA	NA	NA	NA
2	--	BLOD	NA	NA	BLOD
3	--	BLOD	NA	NA	BLOD
4	--	BLOD	NA	NA	BLOD
5	--	BLOD	--	BLOD	BLOD
6	--	BLOD	--	BLOD	BLOD
7	--	BLOD	--	BLOD	BLOD
8	--	BLOD	--	BLOD	BLOD
9	--	BLOD	--	BLOD	BLOD
10	--	BLOD	--	BLOD	BLOD
11	--	BLOD	--	BLOD	BLOD
12	--	BLOD	--	BLOD	BLOD
13	--	BLOD	--	BLOD	BLOD
14	--	BLOD	--	BLOD	BLOD
15	--	BLOD	--	BLOD	BLOD

16	--	BLOD	--	BLOD	BLOD
17	--	BLOD	--	BLOD	BLOD
18	--	BLOD	--	BLOD	BLOD
19	--	BLOD	--	BLOD	BLOD
20	--	BLOD	--	BLOD	BLOD
21	--	BLOD	--	BLOD	BLOD
22	--	BLOD	--	BLOD	BLOD
23	--	BLOD	--	BLOD	BLOD
24	--	BLOD	--	BLOD	BLOD
25	--	BLOD	--	BLOD	BLOD
26	--	BLOD	--	BLOD	BLOD
27	30.1	103.6	--	BLOD	9.2
28	29.2	202.9	28.5	352.6	267.5
29	--	BLOD	31.3	35.2	5.0
30	29.1	61.1	--	BLOD	6.9
31	24.8	1852.4	27.5	412.5	874.4
32	28.6	8949.8	29.0	17.4	405.2
33	28.3	285.1	28.5	210.8	245.2
34	26.3	486.2	26.1	1133.8	742.5
35	26.3	854.2	24.4	3279.8	1674.1
36	24.3	4508.6	28.9	261.5	1087.0
37	24.7	2039.5	26.9	156.9	566.7

38	22.6	8949.8	22.9	6637.5	7707.4
39	21.3	27021.7	23.9	8217.6	14901.6
40	23.8	4854.0	25.0	3712.9	4245.3
41	22.8	15593.0	25.2	4715.8	8575.3
42	26.1	1626.6	27.7	450.1	855.9
43	NA	NA	28.2	299.0	299.0
44	28.4	337.2	23.8	6981.5	1535.8
45	29.6	304.0	--	BLOD	16.5
46	--	BLOD	28.7	316.5	16.8
47	25.2	1803.2	29.8	120.1	466.5
48	--	BLOD	29.1	502.1	21.4
49	--	BLOD	--	BLOD	BLOD
50	31.0	115.2	32.3	42.2	69.8
51	NA	NA	29.9	152.5	152.5
52	NA	NA	--	BLOD	BLOD

WWTP, wastewater treatment plant; PMMoV, pepper mild mottle virus; NA, not available; BLOD, below detection limit.

BLOD indicates that the EV-D68 RNA concentration (the numerator) was below the detection limit before PMMoV normalization.

Table S3. Clinical Characteristics of Patients Positive for Enterovirus D68 on PCR Analysis, in Niigata City, Japan, 2024. (n = 16)

Median age, yr (IQR)	4.8	(3.1–7.5)
Female, n (%)	10	(62.5%)
Past medical history of asthma or wheezing episodes, n (%)	7	(43.8%)
Days from symptom onset to admission, median (IQR)	2.0	(1.8–3.0)
Symptoms other than wheezing		
Tachypnea, n (%)	15	(93.8%)
Cough, n (%)	14	(87.5%)
Retraction, n (%)	14	(87.5%)
Hypoxia (SpO_2 <95%), n (%)	13	(81.2%)
Rhinorrhea, n (%)	11	(68.8%)
High fever (>38.5℃), n (%)	3	(18.8%)
Paralysis, n (%)	0	(0.0%)
Treatment		
Systemic steroids, n (%)	15	(93.8%)
Days of systemic steroids, median (IQR)	4.0	(3.5–5.0)
Supplemental oxygen, n (%)	15	(93.8%)
Days of oxygen use, median (IQR)	3.0	(3.0–4.0)
Admission to intensive care unit	2	(12.5%)
Length of hospital stay, days, median (IQR)	5.0	(4.0–5.3)

IQR, interquartile range
